# New robust subtilisins from halotolerant and halophilic *Bacillaceae*

**DOI:** 10.1007/s00253-023-12553-w

**Published:** 2023-05-09

**Authors:** Fabian Falkenberg, Leonie Voß, Michael Bott, Johannes Bongaerts, Petra Siegert

**Affiliations:** 1 Institute of Nano- and Biotechnologies, Aachen University of Applied Sciences, 52428 Jülich, Germany; 2 Institute of Bio- and Geosciences, IBG-1: Biotechnology, Forschungszentrum Jülich, 52425 Jülich, Germany

**Keywords:** Halotolerant protease, Subtilases, Subtilisin, *Bacillaceae*, Biotechnological application

## Abstract

**Abstract:**

The aim of the present study was the characterisation of three true subtilisins and one phylogenetically intermediate subtilisin from halotolerant and halophilic microorganisms. Considering the currently growing enzyme market for efficient and novel biocatalysts, data mining is a promising source for novel, as yet uncharacterised enzymes, especially from halophilic or halotolerant *Bacillaceae*, which offer great potential to meet industrial needs. Both halophilic bacteria *Pontibacillus marinus* DSM 16465^T^ and *Alkalibacillus haloalkaliphilus* DSM 5271^T^ and both halotolerant bacteria *Metabacillus indicus* DSM 16189 and *Litchfieldia alkalitelluris* DSM 16976^T^ served as a source for the four new subtilisins SPPM, SPAH, SPMI and SPLA. The protease genes were cloned and expressed in *Bacillus subtilis* DB104. Purification to apparent homogeneity was achieved by ethanol precipitation, desalting and ion-exchange chromatography. Enzyme activity could be observed between pH 5.0–12.0 with an optimum for SPPM, SPMI and SPLA around pH 9.0 and for SPAH at pH 10.0. The optimal temperature for SPMI and SPLA was 70 °C and for SPPM and SPAH 55 °C and 50 °C, respectively. All proteases showed high stability towards 5% (w/v) SDS and were active even at NaCl concentrations of 5 M. The four proteases demonstrate potential for future biotechnological applications.

**Key points:**

*• Halophilic and halotolerant Bacillaceae are a valuable source of new subtilisins.*

*• Four new subtilisins were biochemically characterised in detail.*

*• The four proteases show potential for future biotechnological applications.*

**Supplementary Information:**

The online version contains supplementary material available at 10.1007/s00253-023-12553-w.

## Introduction

Microorganisms that can survive in environments with extreme temperatures, pH and salinity produce enzymes called extremozymes (Ferrer et al. 2007). These microorganisms and their enzymes developed molecular mechanisms of adaptation to extreme physico-chemical conditions (Tehei and Zaccai 2005). Of particular interest are proteases, which are one of the most important enzymes used commercially, with subtilisins or alkaline proteases from microbial sources having the largest market share (Gupta et al. 2002; Naveed et al. 2021). Subtilisins are a group of subtilases classified as S8 in the MEROPS database, one of the largest families of serine peptidases (Rawlings et al. 2018). Subtilisins are further subdivided, among others, into true subtilisins, high-alkaline subtilisins, intracellular subtilisins and phylogenetically intermediate subtilisins (PIS) (Siezen and Leunissen 1997; Saeki et al. 2003; Falkenberg et al. 2022a). Especially the genus *Bacillus* proved to be a valuable source of alkaline proteases such as BPN′, subtilisin Carlsberg and Savinase, which are mainly used as detergent enzymes due to their good performance and high stability towards extreme temperatures, pH values, organic solvents, detergents and oxidising agents (Kalisz 1988; Contesini et al. 2017). In addition, subtilisins are used in leather and food processing, sewage purification and as a cosmetic ingredient (Kalisz 1988; Solanki et al. 2021; Azrin et al. 2022).

Enzymes obtained from extremophilic microorganisms are not per se extremozymes in terms of their properties (Ferrer et al. 2007). In environments of extreme pH or salinity, for example, the intracellular enzymes are exposed to conditions more typical of non-extremophiles, as the microorganisms outlast such environments by intracellularly excluding or compensating for such an environment (Ferrer et al. 2007). However, extracellular subtilisins of microbial background are mainly involved in nutrient supply and are therefore directly exposed to the environmental conditions (Kalisz 1988). Therefore, extracellular enzymes isolated from microorganisms found in environments with extreme pH, temperature and especially salinity offer huge potential to meet the needs of industry, as shown by the growing number of newly characterised subtilisins with polyextremophilic properties (Salwan and Sharma 2019; Alberto Cira-Chávez et al. 2019; Coker 2016; Falkenberg et al. 2022b; Mokashe et al. 2018). Besides the labour-intensive search for microorganisms harbouring new enzymes in such extreme environments, genome sequencing and automatic annotation offer an alternative way to search for new protease genes for industrial purposes. Sequence data of uncharacterised proteins are becoming more prevalent due to the growing number of genome sequencing projects (Rawlings 2013). Recently, we reported on a data-mining-based search for new uncharacterised subtilisins from the *Bacillaceae* family (Falkenberg et al. 2022a). Within a phylogenetic tree, these sequences were categorised to the subgroups true subtilisins, PIS and high-alkaline subtilisins. We reported about SPAO from *Alkalihalobacillus okhensis* Kh10-101^T^, which has a high stability towards H_2_O_2_ and NaCl concentrations of up to 5.0 M and belongs to the subgroup of high-alkaline subtilisins (Falkenberg et al. 2022b). Here, we selected three sequences from the phylogenetic tree of true subtilisins and one sequence from the phylogenetically intermediate subtilisins obtained from halotolerant or halophilic bacteria for biochemical characterisation. The true subtilisins (WP_051255158.1, WP_029565418.1, WP_078544469.1) were identified in *Pontibacillus marinus*, *Metabacillus indicus* and *Litchfieldia alkalitelluris* and the PIS WP_146817050.1 in *Alkalibacillus haloalkaliphilus*.

*P. marinus* DSM 16465^T^ is a moderately halophilic bacterium isolated by Lim et al. (2005) from a saline in Korea. The strain is Gram-positive, aerobic and endospore-forming. It grew optimally on media containing 2–5% NaCl (w/v), but did not grow without NaCl or with more than 10% (w/v) NaCl. The optimum growth was observed at pH 7.0–7.5 at 30 °C. *M. indicus* DSM 16189 is a Gram-variable, endospore-forming and halotolerant bacterium isolated by Yoon et al. (2005) from jeotgal, a traditional fermented dish from Korea that contains seafood. The strain was first classified as *Bacillus cibi* in 2005 and then reclassified into *Bacillus indicus* and later into *Metabacillus indicus* (Yoon et al. 2005; Stropko et al. 2014; Patel and Gupta 2020). The strain grew optimally at 37 °C, pH 6.5–7.5 and in the presence of 0–1% (w/v) NaCl, but did not grow with more than 12% (w/v) NaCl (Yoon et al. 2005). The colonies are characteristically orange/yellow pigmented due to the production of carotenoids (Le Duc et al. 2006). *L. alkalitelluris* DSM 16976^T^ is an alkaliphilic bacterium isolated from sandy soil in Korea (Lee et al. 2008). The strain is Gram-positive, endospore-forming and grew optimally at 30 °C and pH 9.0–9.5 (Lee et al. 2008). The strain was reclassified from *Bacillus alkalitelluris* to *Litchfieldia alkalitelluris* (Gupta et al. 2020). The optimal NaCl concentration for growth is 0–1% (w/v), while growth occurs until 4% (w/v) NaCl (Lee et al. 2008). *A. haloalkaliphilus* DSM 5271^T^ was isolated by Weisser and Trüper (1985) from a saline lake of the Wadi Natrun in Egypt. It is a moderate halophilic, Gram-positive, alkaliphilic and spore-forming bacterium. The strain was first classified as *Bacillus haloalkaliphilus* and in 2005 reclassified into *Alkalibacillus haloalkaliphilus* (Fritze 1996; Jeon et al. 2005). It grows at salt concentrations between 1 and 20% (w/v) NaCl with an optimum of 5% (w/v), while cells form a flocculated and slimy sediment without growth in the absence of NaCl (Weisser and Trüper 1985). Optimal growth can be observed at pH 8.5–10.0 and 15–45 °C (Weisser and Trüper 1985; Fritze 1996).

The genes of these four extracellular subtilisins were cloned, overexpressed in *B. subtilis* DB104 and purified. This is the first report on the biochemical characterisation of the recombinant subtilisin proteases of *P. marinus* (SPPM), *M. indicus* (SPMI), *L. alkalitelluris* (SPLA) and *A. haloalkaliphilus* (SPAH).

## Material and methods

### Bioinformatic analysis

The sequence similarity between the four proteases and different well-known characterised subtilisins was investigated within a multiple sequence alignment (MSA) using the peptidase unit sequences from various *Bacillus* strains. The four protein sequences were blasted by using the blastp suite of NCBI (https://blast.ncbi.nlm.nih.gov/Blast.cgi) (Sayers et al. 2021). The signal peptide and propeptide sequences were excluded before alignment and phylogenetic tree construction was performed via *Phylogeny.fr* (http://www.phylogeny.fr/index.cgi) using the “One-Click” option (Dereeper et al. 2008). The signal peptides were identified by the SignalP6.0 software (https://services.healthtech.dtu.dk/service.php?SignalP-6.0) (Teufel et al. 2022). Clustal Omega (https://www.ebi.ac.uk/Tools/msa/clustalo/) was used for MSA before analysis with ESPript 3.0 (Robert and Gouet 2014; Sievers et al. 2011). ESPript 3.0 was applied using %strict option (percentage of strictly conserved residues per column) for the colouring scheme (https://espript.ibcp.fr/ESPript/ESPript/). The phylogenetic tree was visualised with the iTOL software (https://itol.embl.de/) (Letunic and Bork 2016). Structure predictions were performed through the I-TASSER server, including ligand binding prediction with COACH and COFACTOR (https://zhanggroup.org/I-TASSER/) using the amino acid sequence of the peptidase unit of the four proteases (Roy et al. 2012; Yang et al. 2013; Yang et al. 2015). The homology models were displayed with the Mol* Viewer (https://www.rcsb.org/3d-view) (Sehnal et al. 2021). For the determination of the surface-exposed residues and the calculation of the electrostatic potential with the Swiss-PdbViewer (http://www.expasy.org/spdbv/), standard settings using the Poisson-Boltzmann equation were used (Guex and Peitsch 1997). The molecular mass and the theoretical pI of the peptidase unit were determined with the Expasy system (https://web.expasy.org/compute_pi/) (Wilkins et al. 1999).

### Strains and growth conditions

Bacterial strains were bought from the DSMZ—German Collection of Microorganisms and Cell Cultures GmbH and cultivated according to their recommendations: *Pontibacillus marinus* DSM 16465^T^ (Lim et al. 2005) and *Metabacillus indicus* DSM 16189 (Yoon et al. 2005; Stropko et al. 2014; Patel and Gupta 2020) in medium 514 + 10 mg/L MnSO_4_ at 30 °C; *Litchfieldia alkalitelluris* DSM 16976^T^ (Lee et al. 2008; Gupta et al. 2020) in medium 830, pH 9.0 at 30 °C; *Alkalibacillus haloalkaliphilus* DSM 5271^T^ (Weisser and Trüper 1985; Fritze 1996; Jeon et al. 2005) in medium 31 with 5% NaCl, pH 9.7 at 30 °C. For the preparation of genomic DNA from an overnight culture, the InnuSPEED Bacteria/Fungi DNA Kit (Analytik Jena™, Jena, Germany) was used. For cloning and protein production, *Bacillus subtilis* DB104 was used as previously described (Kawamura and Doi 1984; Falkenberg et al. 2022b).

### Plasmid construction and cloning

For recombinant protease production with *B. subtilis* DB104, pFF-RED, a pBC16-based expression plasmid (Acct. No. U32369.1) was used (Bernhard et al. 1978), as described previously (Falkenberg et al. 2022b). The genomic DNA was used to amplify the DNA sequences encoding the protease genes (including signal peptide, propeptide and the peptidase unit) in a PCR using the Phusion® Hot Start II High-Fidelity polymerase (Thermo Fisher Scientific GmbH, Karlsruhe, Germany) according to the manufacturer’s recommendations. The following NCBI reference sequences were used to design primers for the *aprE* genes (extracellular alkaline protease) (Sayers et al. 2021; Clark et al. 2016): NZ_AVPF01000099.1 for *Pontibacillus marinus* DSM 16465^T^ encoding the protein WP_051255158.1; due to an assumed misannotation causing a partial lack of the signal peptide, in this case the annotated ORF was extended by eight codons (24 bp) at the 5′-end, leading to a TTG start codon; NZ_JNVC02000001.1 for *Metabacillus indicus* DSM 16189 encoding the protein WP_029565418.1; NZ_KV917374.1 for *Litchfieldia alkalitelluris* DSM 16976^T^ encoding the protein WP_078544469.1; NZ_BJYA01000014.1 for *Alkalibacillus haloalkaliphilus* DSM 5271^T^ encoding the protein WP_146817050.1. The oligonucleotides listed in Table S1 were obtained from Eurofins Genomics GmbH (Ebersberg, Germany) and used for PCR amplification and the introduction of two BbsI restriction sites and appropriate overhangs to the cloning site of pFF-RED. The PCR products were cloned into the BbsI sites of pFF-RED via Golden Gate cloning (Engler et al. 2008). The resulting plasmids were amplified with rolling-circle mechanism using Illustra TempliPhi 100 amplification kit (Cytiva, Marlborough, USA) before being used to transform naturally competent cells of *B. subtilis* DB104 as described elsewhere (Vojcic et al. 2012). The control of successful cloning and transformation was performed as previously described (Falkenberg et al. 2022b). The production of the protease was confirmed by proteolytic activity assays using N-succinyl-Ala-Ala-Pro-Phe-p-nitroanilide (suc-AAPF-pNA) and azocasein as substrate and by sodium dodecyl sulphate–polyacrylamide gel electrophoresis (SDS-PAGE).

### Recombinant protease production and purification

Production of the proteases by *Bacillus subtilis* DB104 was carried out on a 1-L scale using the DASGIP® parallel reactor system (DASGIP, Jülich, Germany) as described previously (Falkenberg et al. 2022b). The suc-AAPF-pNA assay, azocasein assay and SDS-PAGE were used to confirm the protease production. The protease purification was performed in a three-step protocol as described previously (Falkenberg et al. 2022b). For the proteases an anion exchanger (25 ml Q-Sepharose FF, GE Healthcare, IL, USA) and a pH of 7.0 for running (10 mM HEPES-NaOH buffer) and elution buffer (10 mM HEPES-NaOH, 1 M NaCl) were used.

### Enzyme activity assay

The hydrolytic activity of the proteases was determined using the tetrapeptide substrate suc-AAPF-pNA (BACHEM, Bubendorf, Switzerland) at 30 °C in 100 mM Tris-HCl buffer, pH 8.6, containing 0.1% (w/v) Brij®35 as described previously (DelMar et al. 1979; Falkenberg et al. 2022b). In addition, protease activities were determined using azocasein (Sigma-Aldrich, Schnelldorf, Germany) as substrate according to Brock et al. at 37 °C in 100 mM Tris-HCl buffer, pH 8.6 as described before (Brock et al. 1982; Falkenberg et al. 2022b). The substrate specificity of the proteases for different synthetic 4-nitroanilide substrates was determined as previously described (Falkenberg et al. 2022b).

### Analytical methods, protein measurement and electrophoresis

Protein concentrations were determined according to Bradford (1976) using Roti®Nanoquant (Carl Roth, Germany) and bovine serum albumin fraction V (Carl Roth, Germany) as a standard and measuring the absorbance ratio 590 nm/450 nm. Matrix-assisted laser desorption ionisation-time of flight mass spectrometry (MALDI-TOF-MS) was performed with the Axima confidence instrument (Shimadzu Europe, Duisburg, Germany) as previously described (Falkenberg et al. 2022b). SDS-PAGE analysis was performed as described by Miller et al. (2016) using an 8–20% (v/v) resolving gel and a 6% (v/v) stacking gel. The sample preparation and electrophoresis were performed as described before (Falkenberg et al. 2022b). For isoelectric focusing–polyacrylamide gel electrophoresis (IEF-PAGE), purified proteases were re-buffered in 10 mM HEPES (4-(2-hydroxyethyl)-1-piperazineethanesulfonic acid)-NaOH pH 7.0 using centrifugal spin columns (VWR, Radnor, USA) with a molecular mass cut-off of 3 kDa. The SERVALYT™ PRECOTES™ 3-10 gel (SERVA, Heidelberg, Germany) was used according to the manufacturer’s recommendations.

### Effect of SDS, hydrogen peroxide and PMSF on enzyme activity and stability

SPFA was incubated with H_2_O_2_ (1 and 5% (v/v)) and SDS (1 and 5% (w/v)) in 10 mM HEPES-NaOH, pH 8.0 for 1 h at 10 °C. The influence of the protease inhibitor phenylmethylsulfonyl fluoride (PMSF) was investigated by incubating the proteases in 10 mM HEPES-NaOH with 1 mM PMSF, pH 8.0, for 30 min on ice. Residual activity was measured in the standard suc-AAPF-pNA activity assay and residual activity of the proteases incubated in buffer with no additives was defined as 100%. The stored samples were used undiluted in the activity assay, so that 0.1 and 0.5% of H_2_O_2_ and SDS remained during the measurement.

### Effect of NaCl, CaCl_2_ and EDTA on enzyme activity and stability

The effect of NaCl on proteolytic activity was measured using the suc-AAPF-pNA assay under standard conditions with the addition of NaCl (0–5 M) in the reaction buffer as described before (Falkenberg et al. 2022b). Proteases were incubated in 10 mM HEPES-NaOH pH 8.0 with NaCl (0–5 M) at 20 °C for 2 h to investigate the effects of NaCl on enzyme stability. The activities measured before incubation were defined as 100%. The effect of ethylenediaminetetraacetic acid (EDTA) and CaCl_2_ was examined as described before (Falkenberg et al. 2022b).

### Effects of temperature and pH on enzyme activity and stability

Within a thermal shift assay, the thermal protein unfolding and the melting points of the proteases were determined by using the fluorescent dye SYPRO™ Orange (Thermo Fisher Scientific GmbH, Karlsruhe, Germany) as described previously (Falkenberg et al. 2022b). The temperature optimum was assayed with the suc-AAPF-pNA assay between 20 and 90 °C in 5 °C steps and the temperature stability was determined by measuring the residual activity after incubating the proteases at 20 and 50 °C for 3 h in 10 mM HEPES-NaOH, pH 8.0. The pH optimum of the proteases was determined in 0.1 M Tris-maleate buffer (pH 5.0–7.0), 0.1 M Tris-HCl (pH 7.0–9.0) and 0.1 M glycine-NaOH (pH 9.0–12.5) at 30 °C using the suc-AAPF-pNA assay. Residual activities of the proteases were measured with the suc-AAPF-pNA assay after incubating the proteases in said buffers for 24 h at 4 °C.

## Results

### Cloning and expression of the *aprE* genes in *B. subtilis* DB104

Four uncharacterised proteases were selected from our previous report about a data-mining-based search for new subtilisins from *Bacillaceae* (Falkenberg et al. 2022a). The coding sequences of *aprE_P. marinus* for the protease SPPM, *aprE_M. indicus* for SPMI, *aprE_A. haloalkaliphilus* for SPAH and *aprE_L. alkalitelluris* for SPLA were amplified as described in methods and fragments of 1172 bp, 1152 bp, 1151 bp and 1148 bp were obtained, respectively. The PCR products containing the sequence for the signal peptide, the propeptide and the peptidase unit of the proteases were cloned into the vector pFF-RED and transferred into *B. subtilis* DB104. Successful transformation was checked with arising clearing zones on LB agar plates complemented with 2.5% (w/v) skim milk around the colonies and was analysed by plasmid preparation and restriction analysis. The DNA sequences of the cloned genes were determined by Sanger sequencing and verified that they were identical to the genomic nucleotide sequences of the four *aprE* genes.

### Homology modelling and bioinformatic analysis

**Fig. 1:**
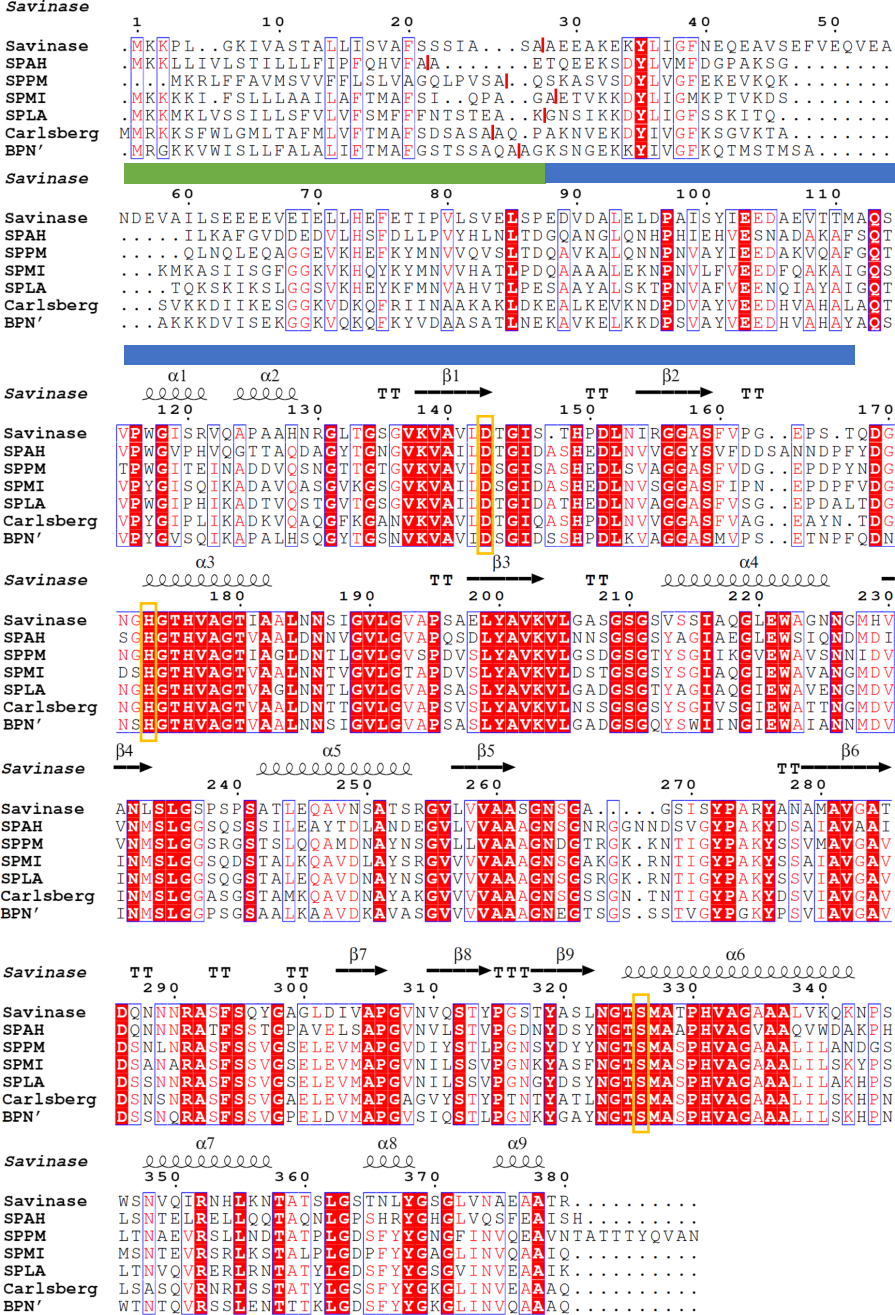
SPPM, SPMI, SPLA, SPAH, Savinase (WP_094423791.1), subtilisin Carlsberg (WP_020450819.1) and BPN′ (WP_013351733.1) within a multiple sequence alignment. The alignment was calculated using Clustal Omega and analysed using ESPript 3.0 and Savinase (PDB: 1C9J) as a template. Solid blue and green bars indicate the propeptide and signal peptide sequence of Savinase. Individual signal peptide cleavage sites are marked with a red bar. Helices are marked with squiggles, β-strands with arrows, and turns with TT letters. The residues of the catalytic triad are marked by orange boxes (Asp^143^, His^173^, Ser^326^; Savinase numbering)

The *aprE* genes from *M. indicus* DSM 16189 and *A. haloalkaliphilus* DSM 5271^T^ comprise 1128 bp each and encode proteins of 375 amino acids. The *aprE* gene from *L. alkalitelluris* DSM 16976^T^ comprises 1131 bp encoding a protein of 376 amino acids. The *aprE* gene from *P. marinus* DSM 16465^T^ comprises 1152 bp encoding a protein of 383 amino acids. In this case, we corrected the automatic annotation by extension of the ORF by eight codons at the 5′-end, leading to a TTG start codon. The signal peptide prediction revealed the presence of a Sec signal peptide for all four selected proteases with a probability above 97% (Fig. 1). The propeptides were identified by multiple sequence alignment and are marked in Fig. 1. The MSA shows that SPAH has a double insertion within a loop between position 42 and 43 in contrast to BPN′ (numbering refers to the mature part of BPN′). In comparison to the other investigated proteases, SPAH has an insertion between position 159 and 160. At the C-terminus, SPPM displays an extension of ten amino acids in comparison to BPN′ and nine amino acids in comparison to SPAH. Without these nine amino acids, the theoretical mass of SPPM is 27.90 kDa, which is congruent to the MALDI-TOF MS analysis as shown later. For the phylogenetic analysis and the homology modelling, the SPPM mature part without its probable C-terminal extension was used. The in silico analysis of mature proteins revealed a molecular mass of 27.48 kDa and a pI of 5.5 for SPMI, 27.47 kDa and a pI of 5.1 for SPLA, 28.6 kDa and a pI of 4.3 for SPAH and 27.90 kDa and a pI of 4.2 for SPPM without the C-terminal extension. The catalytic triad consists of Asp^32^, His^64^ and Ser^221^ in SPPM, SPMI and SPLA and Asp^32^, His^66^ and Ser^224^ in SPAH (numbers based on the mature protease sequences).

**Fig. 2:**
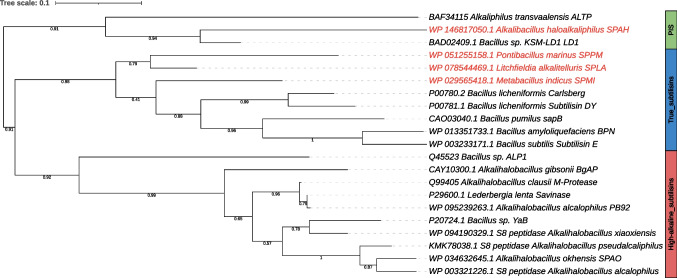
Phylogenetic tree analysis of SPPM, SPMI, SPLA and SPAH with various characterised subtilisins from different species of the family *Bacillaceae*. The Phylogeny.fr server was used for maximum likelihood phylogenetic analysis of the peptidase unit. Branch support is indicated with numbers obtained by approximate likelihood ratio test (SH-like aLRT)

The amino acid sequences of the peptidase unit of the four proteases were aligned and compared in a phylogenetic tree with well-characterised proteases of the three subtilisin families (true, phylogenetically intermediate, high-alkaline) retrieved from the UniProt (The UniProt Consortium 2021) and MEROPS database (Rawlings et al. 2018) as well as our previously characterised high-alkaline subtilisin from *Alkalihalobacillus okhensis* K10-101^T^ (Falkenberg et al. 2022b) (Fig. 2). There, SPAH is clearly a member of the PIS subgroup with a sequence identity of 73.4% to LD1 from *Bacillus* sp. KSM-LD1 (Saeki et al. 2003), 57.7% to ALTP from *Alkaliphilus transvaalensis* (Kobayashi et al. 2007), and a more distant relationship to the well-characterised true subtilisins BPN′ (53.1%) (Matsubara et al. 1965) and the high-alkaline subtilisin Savinase from *Lederbergia lenta* (formerly *Bacillus lentus*) (55.4%) (Betzel et al. 1992). SPPM, SPLA and SPMI cluster together within the subgroup of true subtilisins. The sequence identity between SPPM and SPLA is 76.4% and between SPPM and SPMI 69.8%, while the sequence identity between SPLA and SPMI is 78.6%. The highest sequence identity to the well-characterised subtilisin Carlsberg (Smith et al. 1966) was displayed by SPLA with 74.8%. A more detailed phylogenetic comparison with all subtilisin sequences from *Bacillaceae* was reported before (Falkenberg et al. 2022a).

**Fig. 3:**
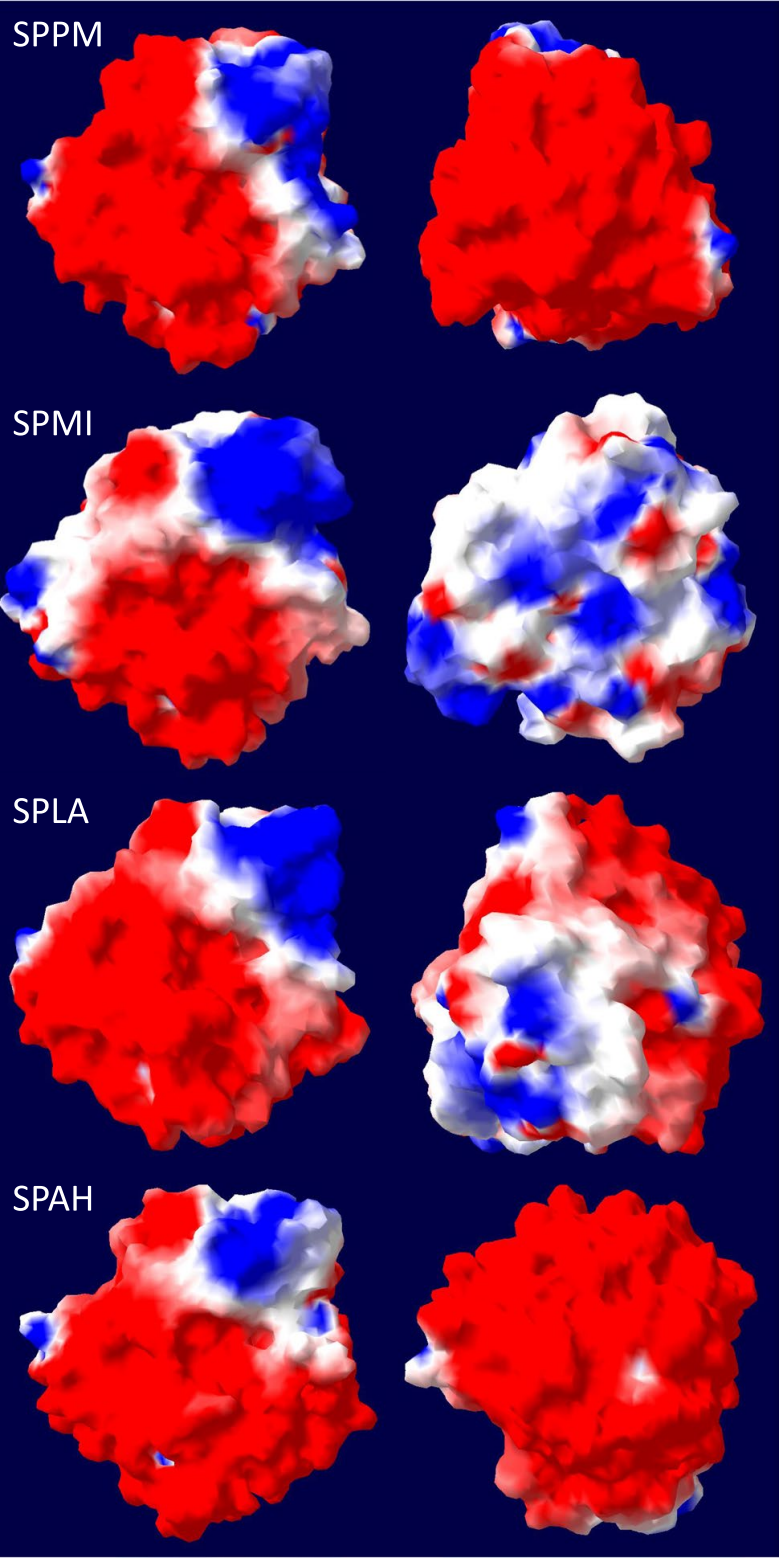
Homology models of SPPM, SPMI, SPLA and SPAH with its calculated protein surface electrostatic potential. Front view of the active site (left); back side of the active site (right). The red (negative) and blue (positive) areas show the electrostatic potential at pH 7.0 calculated with the Swiss-PdbViewer

A homology modelling of the four mature proteases is shown in Fig. S1. The C-score of the models of SPPM and SPMI is 1.52 and of SPLA and SPAH 1.51 and 1.40, respectively. The C-score ranges from −5 to 2 and higher values indicate higher confidence of the model (Zhang 2008). A high TM-score (template modelling) is indicated for BPN′ (PDB: 1S01) with 0.997 for SPPM, 0.995 for SPAH, 0.977 for SPMI and 0.966 for SPLA, where a TM-score of 1 suggests a perfect match between two structures (Zhang and Skolnick 2004). As mentioned above, SPPM contains a presumably nine amino acid long C-terminal extension, and when the 3D structure is calculated with this extension, this extension projects away from the core molecule (data not shown). By using the Swiss-PdbViewer, the 3D structures were used to calculate the electrostatic potential at pH 7.0 as shown in Fig. 3. All four proteases are mainly negatively charged around the active site, while on the back side SPPM and SPAH are completely negatively charged. SPMI shows a more neutral to positive charge, while SPLA is more negatively charged but shows some neutral to positive charged areas at the back side. The in silico analysis of the homology models for Ca^2+^-binding sites suggested that all four proteases harbour two potential Ca^2+^-binding sites. The side chains Gln^2^ and Asp^41^ and several side chains of the loop-forming residues 75–81 including Leu^75^, Asn^77^, Leu^79^ (SPPM and SPLA), Val^79^ (SPMI and SPAH) and Val^81^ (Savinase numbering) are involved in the first Ca^2+^-binding site. The side chains Ala^169^, Tyr^171^, Val^174^ (SPPM and SPLA) and Ala^174^ (SPMI and SPAH) are involved in the second Ca^2+^-binding site.

### Recombinant protease production and purification

**Fig. 4:**
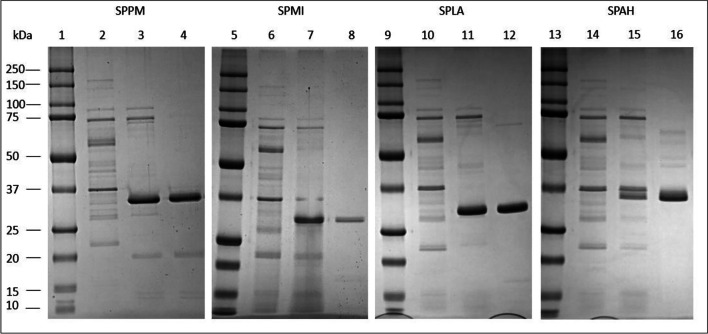
SDS-PAGE analysis of recombinant SPPM, SPMI, SPLA and SPAH. An 8–20% SDS polyacrylamide gel was used for electrophoresis. Bio-Rad Precision Plus Dual Color length marker (1, 5, 9, 13). *B. subtilis* DB104-pFF-RED culture supernatant as negative control (2, 6, 10, 14); *B. subtilis* DB104 culture supernatant producing SPPM (3), after purification (4); SPMI (7, 8), SPLA (11, 12), SPAH (15, 16) before and after purification

Culture supernatants produced from recombinant *B. subtilis* DB104 in a 1-L fermentation showed activity with suc-AAPF-pNA as substrate of 63 U/ml for SPPM, 69 U/ml for SPMI, 73 U/ml for SPLA and 18 U/ml for SPAH. The four supernatants were used for a three-step purification process as described previously (Falkenberg et al. 2022b). The successful purification of the four proteases to apparent homogeneity was confirmed via SDS-PAGE (Fig. 4). The proteases SPPM and SPAH migrate at approximately 35 kDa in contrast to the theoretical molecular mass of 27.9 and 28.6 kDa, respectively. SPMI and SPLA migrate also higher than the theoretical molecular mass of 27.5 kDa at approximately 30 kDa. The MALDI-TOF MS analysis revealed a molecular mass of 27.49 kDa for SPMI, 27.48 kDa for SPLA, 28.60 kDa for SPAH and 27.97 kDa for SPPM (Fig. S2).

Purified SPPM and SPMI had specific activities of 208 and 160 U/mg for the suc-AAPF-pNA substrate and 1371 and 1085 U/mg for azocasein, respectively. Purified SPLA and SPAH had a specific activity of 233 and 314 U/mg for the AAPF substrate and 1036 and 2719 U/mg for azocasein. The analysis of the isoelectric point of the purified and rebuffered proteases showed a pI for SPPM of approx. 4.3, which is near the predicted pI of 4.2 (Fig. S5). For SPMI, a pI of approx. 5.5 was measured, which corresponds to the theoretical value. Furthermore, SPLA showed a pI of approx. 5.0, close to the theoretical pI of 5.1, and SPAH had a pI of approx. 4.9, which deviates from the theoretical pI of 4.3. All four proteases have an acidic pI and an AB ratio above 1.0 with a high number of Asp residues (Table S2).

### Effects of temperature and pH on enzyme activity and stability

**Fig. 5:**
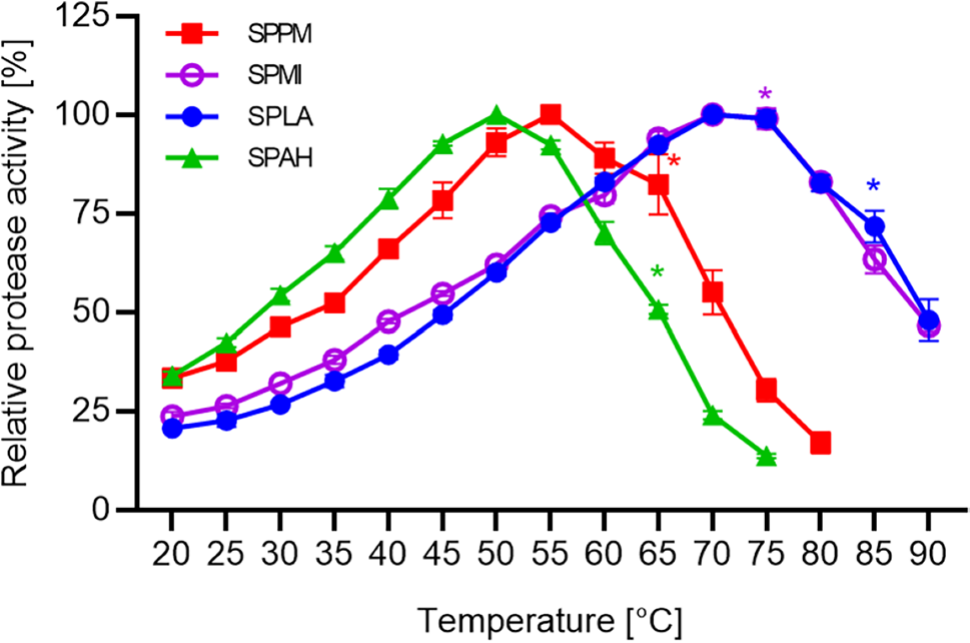
Activity of purified SPPM, SPMI, SPLA and SPAH at different temperatures. Protease activity at temperatures between 20 and 90 °C was measured with the suc-AAPF-pNA assay. Maximum activity for each protease was defined as 100%: 263 U/mg for SPPM (red squares), 540 U/mg for SPMI (violet open circles), 1092 U/mg for SPLA (blue circles) and 371 U/mg for SPAH (green triangles). *The enzyme was not stable for the intended 5 min. The experiments were performed in triplicates and data are plotted as means ± SD

The influence of temperature on enzyme activity was investigated in a temperature range from 20 to 90 °C at a pH of 8.6, as described in the methods section (Fig. 5). The activity of SPLA and SPMI gradually increased from 20 °C to the optimum of 70 °C and decreased to 48 and 47% residual activity at 90 °C, respectively. The lowest temperature optimum of 50 °C showed SPAH, and activity measurement was only possible up to 75 °C with residual activity of 14%. The temperature profile is comparable to that of SPPM with an optimum at 55 °C and 17% residual activity at 80 °C.

**Fig. 6:**
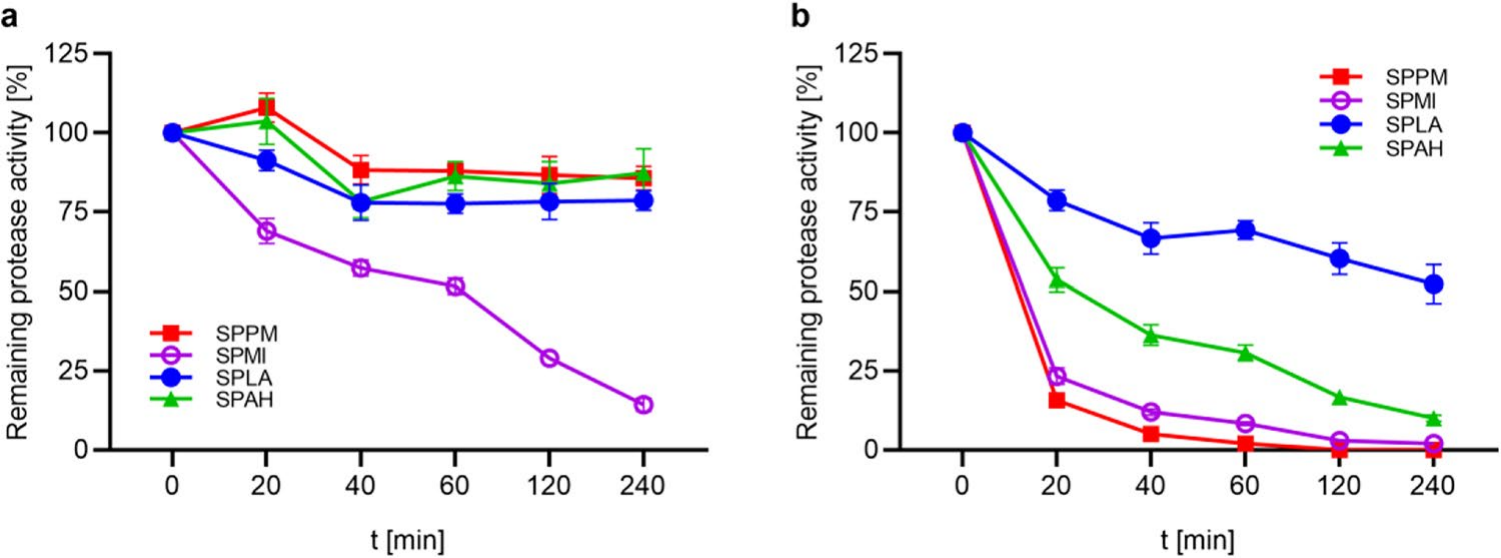
Temperature stability of purified SPPM, SPMI, SPLA and SPAH. Stability was studied at 20 °C (**a**) and 50 °C (**b**) in 10 mM HEPES-NaOH buffer, pH 8.0. The suc-AAPF-pNA assay was used to measure the activity at 30 °C. The activity at 0 min was defined as 100%: 121 U/mg for SPPM (red squares), 151 U/mg for SPMI (violet open circles), 212 U/mg for SPLA (blue closed circles) and 197 U/mg for SPAH (green triangles). The experiment was performed in triplicates and data are plotted as means ± SD

Enzyme stability towards different temperatures was investigated either by incubation at 20 and 50 °C or by monitoring thermal protein unfolding in a thermal shift assay. Figure 6 shows the remaining protease activity during an incubation at 20 and 50 °C for 4 h. The activity of SPPM, SPLA and SPAH remained quite stable with a residual activity of over 75% after 4 h, while SPMI lost 85% of its activity in this period. The loss of activity was more distinct during incubation at 50 °C. While SPPM and SPMI lost all their remaining activity after 4 h at 50 °C, SPAH retained 10% and SPLA 52% of their activity. However, a comparison of the temperature stability of proteases is difficult due to the possible autoproteolysis during incubation. In order to monitor thermal protein unfolding instead of autoproteolysis, the proteases were tested in a thermal shift assay. Proteases were inhibited with phenylmethylsulfonyl fluoride (PMSF) and denaturation curves were recorded (Fig. S4). SPMI and SPLA revealed melting points (T_m_) of 62.5 and 61.5 °C, respectively. It was not possible to obtain a melting curve for SPPM and SPAH.

**Fig. 7:**
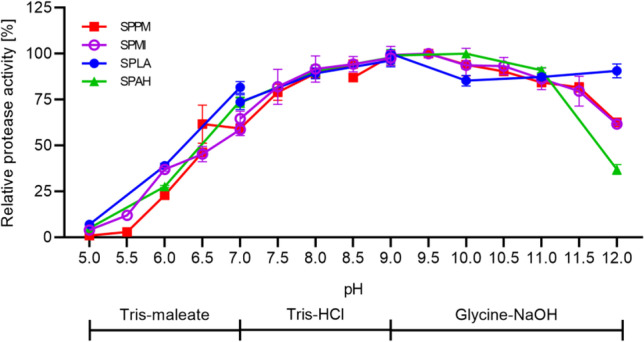
SPPM, SPMI, SPLA and SPAH activity at different pH. The test was performed with the suc-AAPF-pNA assay in the pH range of 5.0–12.0 at 30 °C. Maximum activity for each protease was defined as 100%: 98 U/mg for SPPM (red squares), 104 U/mg for SPMI (violet open circles), 201 U/mg or SPLA (blue closed circles) and 176 U/mg for SPAH (green triangles). The experiments were performed in triplicates and data are plotted as mean values ± SD

At a pH range of 5.0–12.0 showed all four proteases a comparable pH profile until pH 11.0 (Fig. 7). For SPPM and SPMI, the highest activity was observed at pH 9.0–9.5. The relative activities at pH 5.0 and 12.0 were 1% and 63% for SPPM and 4% and 62% for SPMI. SPLA and SPAH showed pH optima at pH 9.0 and 10.0, respectively. The relative activities at pH 5.0 and 12.0 were 7% and 91% for SPLA and 5% and 37% for SPAH. The stability test of the proteases at different pH values showed that all four proteases retained a residual activity of at least 65% at pH 5.0, 94% around the pH optimum and 83% at pH 12.0 (Fig. S3).

### Effect of SDS and H_2_O_2_ on enzyme activity

The activity of the four proteases after 1-h incubation with 1 and 5% SDS (w/v) at 10 °C showed that they possess a high stability towards SDS (Table 1). All proteases exhibited an enhanced activity after incubation with 1% SDS of up to 178% (SPLA). After incubation with 5% SDS, only SPPM revealed a reduced residual activity of 76%, while SPLA with 97% residual activity showed almost no decrease. SPMI displayed the highest stability and had a residual increased activity of 165% after incubation with 5% SDS.

**Table 1:** Influence of SDS, H_2_O_2_ and PMSF on enzyme activity

Protease	Residual protease activity (%)				
	1% SDS	5% SDS	1% H_2_O_2_	5% H_2_O_2_	1 mM PMSF
SPPM	121 ± 4	76 ± 2	92 ± 4	41 ± 0	0 ± 0
SPMI	178 ± 4	165 ± 1	81 ± 1	31 ± 2	0 ± 0
SPLA	108 ± 4	97 ± 3	78 ± 2	40 ± 1	0 ± 0
SPAH	120 ± 7	106 ± 2	89 ± 1	40 ± 2	0 ± 0

The influence of 1 and 5% (v/v) H_2_O_2_ is shown in Table 1. After 1 h of treatment with 1% H_2_O_2_, all proteases showed a high residual activity of 78 to 92%. Treatment with 5% H_2_O_2_ reduced the remaining activity to 31–40%. PMSF is a classical inhibitor for serine proteases (North 1982), and the incubation of the four serine proteases SPPM, SPMI, SPLA and SPAH with 1 mM PMSF led to a complete inhibition (Table 1).

The purified proteases were incubated with 1 and 5% (v/v) H_2_O_2_, 1 and 5% (w/v) SDS and 1 mM PMSF in 10 mM HEPES-NaOH pH 7.0 for 1 h at 10 °C. Remaining activity of the proteases incubated in buffer with no additions was defined as 100%. During the measurement, 0.1 and 0.5% of H_2_O_2_ and SDS remained, respectively. All experiments were performed at least in triplicates and data are shown as mean values ± SD

### Effect of NaCl, CaCl_2_ and EDTA on enzyme activity and stability

**Fig. 8:**
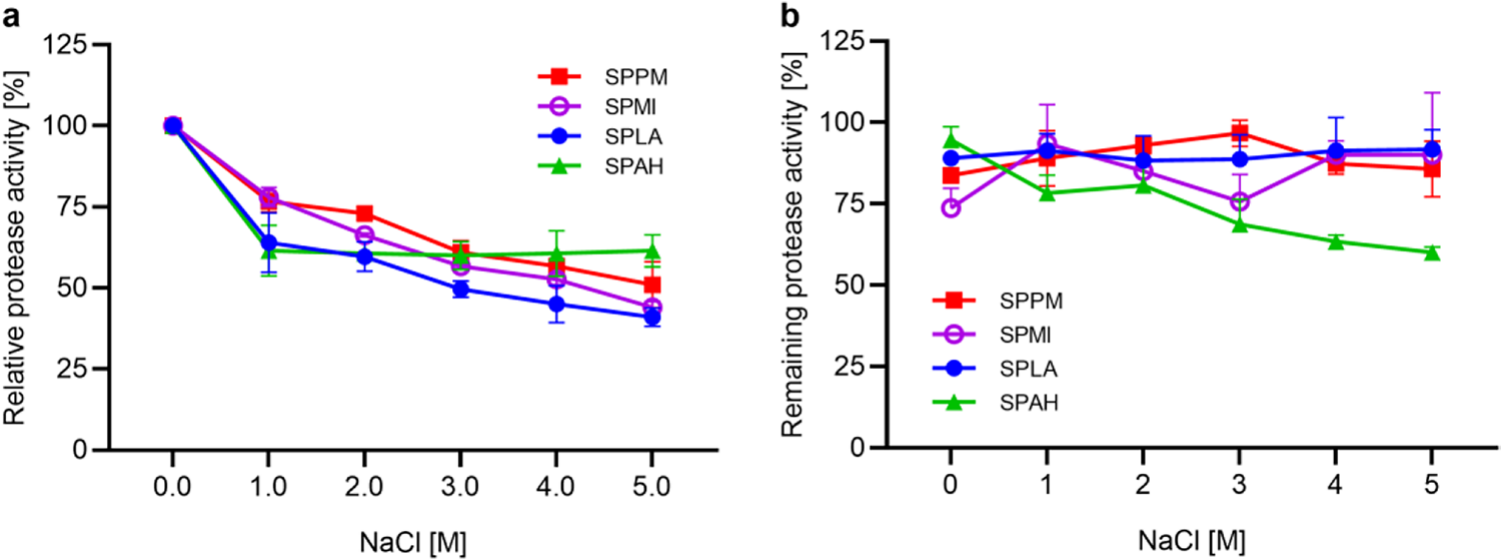
The effect of NaCl on the activity (**a**) and stability (**b**) of the purified proteases SPPM, SPMI, SPLA and SPAH. Experiment was performed with the suc-AAPF-pNA assay at 30 °C with different NaCl concentrations (0–5 M). Maximum activity for each protease was given as 100%: 551 U/mg for SPPM, 601 U/mg for SPMI, 337 U/mg for SPLA and 207 U/mg for SPAH. The proteases were incubated in 10 mM HEPES-NaOH buffer, pH 8.0, with the indicated NaCl concentrations to test the stability. Residual activity was assayed after 2-h incubation at 20 °C with the suc-AAPF-pNA assay in standard buffer at pH 8.6. The initial activity at each NaCl concentration was considered as 100% activity. The experiments were performed in triplicates and data are given as mean values ± SD

In the suc-AAPF-pNA activity assay with different NaCl concentrations (0–5 M), the protease SPPM showed the highest activity without NaCl and the activity gradually decreased with higher NaCl concentrations to 51% at 5 M NaCl, comparable to the proteases SPMI and SPLA with 44 and 41% residual activity (see Fig. 8a). The protease SPAH revealed the highest activity at 0 M NaCl, which dropped to 60% at 1 M NaCl, but then displayed no further loss at higher NaCl concentrations. In addition, the stability of the proteases was investigated in the presence of salt by incubation with NaCl (0–5 M) in 10 mM HEPES-NaOH, pH 8.0 for 2 h at 20 °C (Fig. 8b). SPPM, SPMI and SPLA were stable with and without NaCl, while SPAH lost activity with increasing NaCl concentration.

The effect of Ca^2+^ on the activity of the proteases was studied as subtilisins are calcium-dependent and the binding of Ca^2+^ is essential for enzyme activity and stability (Siezen et al. 1991). The in silico studies revealed two calcium binding sites for the four proteases, as mentioned above. When incubated with or without EDTA, SPPM and SPAH showed almost no difference in activity (data not shown). Recovery of activity was not possible for SPLA and SPMI after the addition of CaCl_2_.

### Proteolytic activity on synthetic peptides

The specificity of the four proteases towards ten synthetic peptide 4-nitroanilide substrates with three or four amino acids was analysed (Table 2). With the exception of suc-AAA-pNA, which is an elastase substrate, all are typical subtilisin substrates (Georgieva et al. 2001; Bieth et al. 1974). The proteases showed a very low specificity for suc-YVAD-pNA, suc-TVAA-pNA, suc-AAA-pNA and suc-AAVA-pNA. Highest activity was displayed for suc-ALPF-pNA and suc-AGPP-pNA.

**Table 2:** Substrate specificities of the indicated proteases against 10 synthetic substrates (suc-XXXX-pNA)

Protease	Relative activity (%)									
	FAAF	AAA	AAVA	ALPF	AGPF	AAPF	TVAA	YVAD	AGPP	AAPL
SPPM	25	1	3	140	97	100 (190 U/mg)	1	2	127	78
SPMI	44	1	3	139	88	100 (223 U/mg)	1	1	146	68
SPLA	26	1	3	130	100	100 (93 U/mg)	1	2	173	73
SPAH	32	1	3	140	102	100 (159 U/mg)	1	1	157	67
SPAO^a^	753	12	21	127	107	100 (42 U/mg)	3	3	216	34
Subtilisin Carlsberg^a^	57	0	2	60	90	100 (570 U/mg)	1	1	147	104
Savinase^a^	605	8	22	117	96	100 (180 U/mg)	5	5	144	12
BPN′^a^	96	0	6	106	96	100 (181 U/mg)	0	0	61	67

Kinetic experiments were performed with 17 mM substrate at 30 °C in 0.1 M Tris-HCl buffer pH 8.6 containing 0.1% (w/v) Brij®35 for 5 min. The experiments were performed in triplicates and the standard deviation was <5%. Enzyme activity against AAPF was defined as 100% relative activity

^a^Falkenberg et al. (2022b)

## Discussion

Subtilisins are extremely versatile serine peptidases from the subtilase family and due to their properties such as thermostability, broad pH range and broad specificity, of particular interest for industrial applications (Azrin et al. 2022). Especially subtilisins from halophilic or halotolerant *Bacillaceae* have a high potential for meeting industrial needs (Salwan and Sharma 2019; Alberto Cira-Chávez et al. 2019; Coker 2016). Therefore, in this study, we characterise four subtilisins from halophilic and halotolerant *Bacillaceae* found in our previous publication through a data mining search (Falkenberg et al. 2022a).

The coding sequences of *aprE_P. marinus* for the protease SPPM, *aprE_M. indicus* for SPMI, *aprE_A. haloalkaliphilus* for SPAH and *aprE_L. alkalitelluris* for SPLA were amplified. Most subtilases consist of a signal peptide for translocation, a propeptide for maturation, a protease domain and sometimes additional domains (Siezen et al. 2007). However, the annotated gene of SPPM would lack eight amino acids of the signal peptide. In this case, we corrected the automatic annotation by extension of the ORF by eight codons at the 5′-end, leading to a TTG start codon, which is not uncommon for *Bacillus* sp. (Rocha et al. 1999).

When analysing the amino acid sequence of the four proteases in a multiple sequence alignment, shows that SPAH has a double insertion within a loop between position 42 and 43, and an insertion between position 159 and 160 in contrast to BPN′. These insertions are typical of phylogenetically intermediate subtilisins, as our earlier study shows (Falkenberg et al. 2022a). Additionally, around position 160 high-alkaline subtilisins have a four-amino acid deletion in common (Falkenberg et al. 2022a), which cannot be observed for SPPM, SPMI, SPLA and SPAH. Position 160 is within a loop associated with the P1 binding site, which is the first position N-terminal to the cleavage site and therefore may be involved in P1 preference and steric conformation (Wells et al. 1987; Betzel et al. 1992; Yamagata et al. 1995; Schechter and Berger 1967). In addition, shorter loops can increase enzyme stability (Gavrilov et al. 2015).

Furthermore, the subtilisins of *Bacillus* species usually contain one strong and one weak Ca^2+^-binding site (Siezen and Leunissen 1997). The strong Ca^2+^-binding site, which is conserved in diverse subtilases, requires the side chains of residues Gln^2^ and Asp^41^ and several side chains of the loop-forming residues 75–81, which are also found for the four proteases (Siezen and Leunissen 1997; Betzel et al. 1992). The occupancy of the weak site is dependent of the CaCl_2_ concentration in the solution and the side chains Ala^169^, Tyr^171^, Val^174^ (SPPM and SPLA) and Ala^174^ (SPMI and SPAH) are involved (Savinase numbering) (Siezen et al. 1991; Betzel et al. 1992). When the proteases were incubated with EDTA, SPPM and SPAH showed nearly no difference in activity between incubation with and without EDTA. SPLA and SPMI, however, lost activity. Since Ca^2+^ has a stabilising effect on the protease, this could be due to a general loss of activity during the incubation with EDTA (Siezen et al. 1991), which was also observed by others (Dodia et al. 2008; Thaz and Jayaraman 2014; Vidyasagar et al. 2009). A protein-engineered version of BPN′ led to a calcium-independent protease that is fully active, but has a lower thermal stability in the absence of stabilising mutations (Almog et al. 1998).

SPPM, SPMI, SPLA and SPAH migrate higher on the SDS-PAGE than would be expected on the basis of the theoretical masses. A migration behaviour of proteins during SDS-PAGE deviating from the expected molecular mass is not uncommon and may occur due to partial refolding or altered detergent binding (Matagne et al. 1991; Rath et al. 2009). The MALDI-TOF-MS analysis thus confirmed the theoretical masses. SPPM displays an extension of ten amino acids in comparison to BPN′ and nine amino acids in comparison to SPAH at the C-terminus when comparing the amino acid sequences. Without these nine amino acids, the theoretical mass of SPPM is 27.90 kDa. A mass of 27.97 kDa was determined by MALDI-TOF MS, close to the value of 27.90 kDa predicted for the variant lacking the C-terminal nine amino acids. Therefore, the C-terminus seems to be processed in an intermolecular process by other SPPM molecules, as this extension projects away from the core molecule, supporting the assumption that it is prone to proteolytic processing. However, it could also be that only 8 amino acids are cleaved off, because with the additional Ala, the calculated molecular weight would agree with the experimental one.

The four proteases have a temperature optimum of 50–70 °C, which is in the range of other subtilisins such as subtilisin Carlsberg, BPN′ and Savinase, which have their optimum at 65 °C, 55 °C and 60 °C, respectively (Falkenberg et al. 2022b). Comparing the optimal temperatures with the optimal growth temperature of the bacterial origins, differences of up to 20 °C can be observed. *P. marinus* and *L. alkalitelluris*, the native hosts of SPPM and SPLA, grow between 15 and 40 °C (Lim et al. 2005; Lee et al. 2008). *M. indicus* and *A. haloalkaliphilus*, the native hosts of SPMI and SPAH, grow at 4–50 °C and 15–45 °C, respectively (Yoon et al. 2005; Fritze 1996). This observation is consistent with the study of Engqvist (2018) who reported that proteins from mesophiles (15–50 °C) tend to be catalytically active at higher temperatures than expected based on the growth temperature. When analysing the melting point of the four proteases, it was not possible to obtain a melting point for SPPM and SPAH. According to Boivin et al., this could be due to protein precipitation, aggregation, some intrinsically disordered regions with complicated folding landscape or a high hydrophobic background masking the melting transition (Boivin et al. 2013). The high melting point of SPMI and SPLA compared to the value obtained for the subtilisin SPAO from *Alkalihalobacillus okhensis* (T_m_ of 53.0 °C) correlates with the higher temperature optimum (Fig. 5) (Falkenberg et al. 2022b).

Although the proteases do not belong to the high-alkaline proteases phylogenetically, they showed a high activity at alkaline pH until pH 12.0, which shows the potential for various industrial applications (Tekin et al. 2021; Phrommao et al. 2011; Gurunathan et al. 2021). In particular, SPLA is characterised by a high residual activity of 91% at pH 12.0, which is even higher than the residual activity of 53% of the high-alkaline subtilisin Savinase (Falkenberg et al. 2022b). The two other true subtilisins SPPM and SPMI with a residual activity of over 60% also stand out, as other proteases within this subgroup such as BPN′, subtilisin Carlsberg and endopeptidase Q show lower activity with 6, 19 and about 8% relative residual activity at pH 12.0, respectively (Falkenberg et al. 2022b; Han and Damodaran 1998). In contrast to SPAH with a residual activity of 37%, the phylogenetically intermediate subtilisin ALTP from *Alkaliphilus transvaalensis* showed its pH optimum at a pH above 12.6 (Kobayashi et al. 2007). The adaptation to higher alkaline conditions by high-alkaline subtilisins is indicated by an altered surface charge with an increased pI value and, in particular, by an increased amount of Arg and a reduced amount of Lys residues (Masui et al. 1998). Interestingly this correlation does not hold with the four investigated proteases, as they all have an acidic pI and, compared to the two high-alkaline subtilisins SPAO and Savinase, a decreased number of Arg residues and an increased number of Asp residues (Falkenberg et al. 2022b). An increased number of charged amino acids on the protein surface leads to better ionic interactions, thus maintaining stability and solubility (Panja et al. 2020). The stability test of the proteases at different pH values showed that all four proteases retained a residual activity of at least 65% at pH 5.0, 94% around the pH optimum and 83% at pH 12.0 (Fig. S3). This is comparable to other subtilisins as previously discussed (Falkenberg et al. 2022b).

When incubated with 1 and 5% SDS (w/v), SPPM, SPMI, SPLA and SPAH all show high stability towards SDS. As reported in our previous study, the three known proteases subtilisin Carlsberg, BPN′ and Savinase also showed higher activities after incubation with 1 and 5% (w/v) SDS than without (Falkenberg et al. 2022b). Instead of supporting protein unfolding, SDS can help to achieve a favourable protein conformation in some subtilisins, as has been reported for other subtilisins as well (Falkenberg et al. 2022b; Bhatt and Singh 2020; Joshi and Satyanarayana 2013; Thebti et al. 2016). For a salt-tolerant and thermostable protease from *B. subtilis*, no loss of activity was observed even at an SDS concentration of 10% (w/v) (Kembhavi et al. 1993). Rekik et al. reported for an alkaline serine protease from *Bacillus safensis* RH12 a reduction of the residual activity to 90 and 60% after incubation with 1 and 5% SDS (Rekik et al. 2019). However, for SPAO from *A. okhensis*, incubation with SDS led to a complete loss of activity, which is quite unusual for highly-alkaline subtilisins (Falkenberg et al. 2022b).

When analysing stability against H_2_O_2_, the four new proteases are highly stable against oxidation, contrary to Savinase, subtilisins Carlsberg and BPN′, which lost up to 92% of their activity (Savinase) under these conditions (Falkenberg et al. 2022b). However, our previously reported high-alkaline subtilisin SPAO showed an even higher resistance to H_2_O_2_ with an increased activity of 108% at 1% H_2_O_2_ and a remaining activity of 58% after incubation with 5% H_2_O_2_ (Falkenberg et al. 2022b). The sensitivity to H_2_O_2_ is probably due to a conserved methionine near the catalytic serine, which is oxidised to its sulphoxide. The sulphoxide oxygen is directed towards the oxyanion hole and destabilises the tetrahedral intermediate formed with the carbonyl group of the substrate (Stauffer and Etson 1969; Bott et al. 1988; Bryan et al. 1986). The comparison of the obtained data with literature data is difficult due to the different experimental conditions. Subtilisin LD1 from *Bacillus* sp. KSM-LD1 maintained 40% of its activity after incubation with 3.4% (v/v) H_2_O_2_ at 30 °C for 30 min, and a protease from *Bacillus patagoniensis* was not affected by H_2_O_2_ (10% v/v) after incubation for 30 min at 25 °C (Olivera et al. 2006; Saeki et al. 2003).

With regard to tolerance and activity under saline conditions, it is interesting to look at the bacterial origin. *A. haloalkaliphilus*, the native host of SPAH, grows at salt concentrations between 1 and 20% with an optimum of 5%, while *P. marinus*, the origin of SPPM, grows between 1 and 9% NaCl with an optimum between 2 and 5% (Lim et al. 2005; Weisser and Trüper 1985). SPMI and SPLA derive from halotolerant bacteria, with *L. alkalitelluris* growing between 0 and 4% NaCl and *M. indicus* between 0 and 12% NaCl. Both species show an optimum between 0 and 1% NaCl (Lee et al. 2008; Yoon et al. 2005). Interestingly, the proteases are still active at NaCl concentrations at which the bacterial strains no longer grow. The highest activity was found for all proteases without NaCl, while for the two halophilic strains at least 1% NaCl is required for growth. The PIS enzyme SPAH maintained its activity after an initial loss of activity, whereas the three true subtilisins SPMI, SPPM and SPLA constantly lost activity. The activity of the previously investigated high-alkaline subtilisins is even induced by high salt concentrations (Falkenberg et al. 2022b). In contrast to the true subtilisins studied here, BPN′, which belongs to the same subgroup, showed higher activity with increasing NaCl levels (Falkenberg et al. 2022b). This is probably because BPN′ has five of the seven amino acid positions identified that are beneficial for salt adaptation (Takenaka et al. 2022). The finding that SPAH lost more activity at higher NaCl concentrations could be related to the constant activity at high salt concentrations and thus a higher autoproteolytic activity, which was also observed for subtilisin Carlsberg (Falkenberg et al. 2022b). No loss of activity at up to 5 M NaCl was also reported for a salt-tolerant and thermostable protease from *B. subtilis* (Kembhavi et al. 1993). Although the mechanism of halotolerance is not yet fully understood, a charged surface of the protein leads to increased hydration of the enzyme surface, which provides protection against aggregation at high salt concentrations (Mokashe et al. 2018; Takenaka et al. 2018). In addition to predicting adaptation to higher pH values by looking at the charge on the protein surface, this can also be used to predict salt tolerance. This is indicated by an acidic isoelectric point and the ratio of glutamate, aspartate to lysine, histidine and arginine (AB ratio) (Rhodes et al. 2010; Mokashe et al. 2018). All four proteases have an acidic pI and an AB ratio above 1.0 with a high number of Asp residues (Table S2). Whereas the surface charge of high-alkaline subtilisins is predominantly positive, it is predominantly negative for the four proteases of this study (Fig. 3). As mentioned above, salt adaptation is increased by a high number of negative or positive charges on the surface of the enzyme (Takenaka et al. 2022). The differences in the AB ratio of all residues seems to have no influence on the salt tolerance, as the AB ratio of SPPM (1.9) and SPAH (1.7) is quite high, but much lower for SPMI (1.0) and SPLA (1.1) (Fig. 3, Table S2).

Most subtilisins have broad substrate specificity and mainly have a nutritional role by supplying peptides and amino acids for cell growth (Siezen and Leunissen 1997). Variations in substrate specificity occur due to modulations of residues in the substrate-binding region, especially whose side chains interact with substrate residues P1 and P4, which dominate substrate preference in subtilisins (Siezen and Leunissen 1997; Grøn et al. 1992). The use of ten synthetic peptide 4-nitroanilide substrates with three or four amino acids makes it possible to compare the preference with others. As we have previously reported, the high-alkaline subtilisin SPAO and Savinase revealed the highest activity for suc-FAAF-pNA, while BPN′ preferred suc-ALPF-pNA and the subtilisin Carlsberg suc-AGPP-pNA, which is comparable to the preferences of the proteases from this work (Falkenberg et al. 2022b). Georgieva et al. showed for several subtilisins and proteinase K that they show lower activity when alanine, glutamate, lysine or valine are in position P1 nomenclature of Schechter and Berger (1967) (Georgieva et al. 2006). Based on the substrate specificity, SPPM, SPMI, SPLA and SPAH can be regarded as typical subtilisins.

In summary, we describe the production, purification and biochemical characterisation of the four extracellular subtilisin proteases SPPM, SPMI, SPLA and SPAH. The sequences were obtained from a data-mining search for new subtilisins from *Bacillaceae*, as this family has proven to be a valuable source of alkaline proteases with industrial applications. The genes were isolated from the two halophilic bacteria *P. marinus* DSM 16465^T^ and *A. haloalkaliphilus* DSM 5271^T^ and the two halotolerant species *M. indicus* DSM 16189 and *L. alkalitelluris* DSM 16976^T^. The proteases showed high halotolerance up to 5 M NaCl and activity within a broad pH spectrum of pH 5.0–12.0 with an optimum between pH 9.0–10.0. The optimum temperature was found to be 50 and 55 °C for SPAH and SPPM and 70 °C for SPMI and SPLA. In addition, a high stability towards 5% (w/v) SDS and a good stability towards 5% (v/v) H_2_O_2_ were observed. With their biochemical properties, the four proteases show the potential for future biotechnological applications and that bacteria of halotolerant or halophilic origin are a promising source for novel enzymes.

## Supplementary information

ESM 1

## Data Availability

The original contributions presented in this study are included in the article/Supplementary material; further inquiries can be directed to the corresponding author.
