# Supplementary material for: New robust subtilisins from halotolerant and halophilic Bacillaceae
Source: Appl Microbiol Biotechnol. 2023 May 9;107(12):3939–54. doi: 10.1007/s00253-023-12553-w (PMC10238314; doi:10.1007/s00253-023-12553-w)
Supplement: Supplementary file 1 — ESM 1 [file 253_2023_12553_MOESM1_ESM.pdf]

Title:

# New robust subtilisins from halotolerant and halophilic *Bacillaceae*

Journal name:

Applied Microbiology and Biotechnology

Author's names:

Fabian Falkenberg[1], Leonie Voß[1], Michael Bott[2], Johannes Bongaerts[1], Petra Siegert[1]

Addresses:

1 Institute of Nano- and Biotechnologies, Aachen University of Applied Sciences, 52428, Jülich, Germany.
2 Institute of Bio- and Geosciences, IBG-1: Biotechnology, Forschungszentrum Jülich, 52425 Jülich, Germany.

Corresponding author:

P. Siegert, Institute of Nano- and Biotechnologies, Aachen University of Applied Sciences, 52428, Jülich, Germany
Tel.: +49 241 6009 53124
E-mail: siegert@fh-aachen.de
**https://www.fh-aachen.de/forschung/inb**

# Supplementary

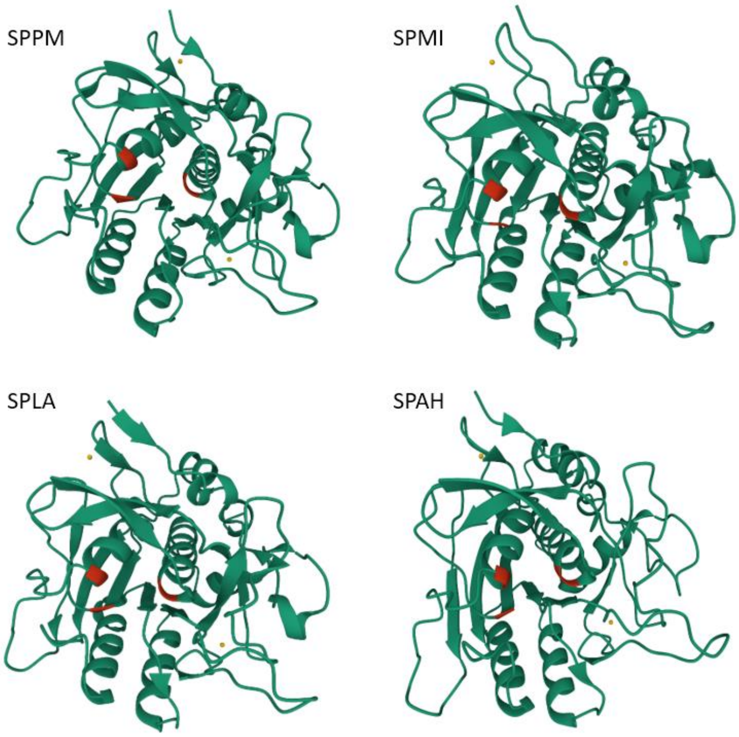


**Fig. S1** Homology models of the mature forms of SPPM, SPMI, SPLA and SPAH obtained using I-TASSER software. *In silico* metal-binding analysis predicted the existence of two $Ca^{2+}$-binding sites (yellow balls). The catalytic residues $Asp^{32}$, $His^{64}$, and $Ser^{221}$ for SPPM, SPMI, SPLA and $Asp^{32}$, $His^{66}$, and $Ser^{224}$ for SPAH are shown in red

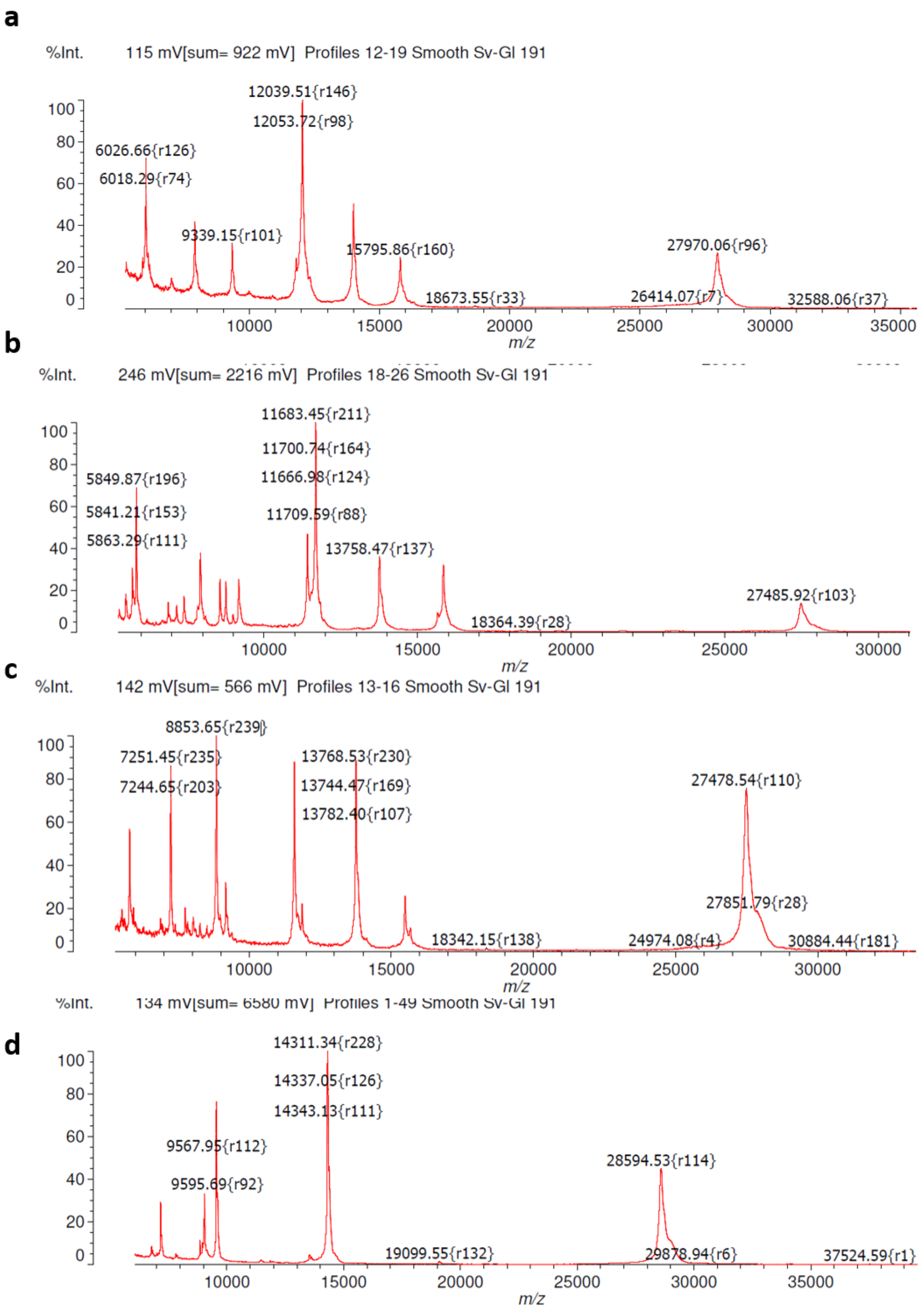


**Fig. S2** MALDI-TOF mass spectra of SPPM (a), SPMI (b), SPLA (c) and SPAH (d). The labels on the peaks indicate the measured average molecular mass. The peaks correspond from right to left M/z up to M/5z

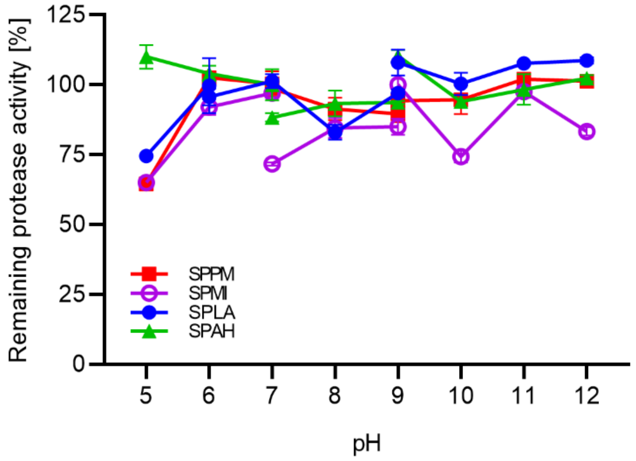


**Fig. S3** The effect of pH on the stability of purified SPPM, SPMI, SPLA and SPAH. The activity was measured with the suc-AAPF-pNA assay in standard buffer at pH 8.6 after incubation for 4 h at 4 °C in Tris-maleate buffer (pH 5 – 7), in Tris-HCl (pH 7 – 9), and in glycine-NaOH (pH 9 – 12). The activity at 0 h was considered as 100 %; highest residual activities: 115 U/mg for SPPM (red squares), 165 U/mg for SPMI (violet open circles), 219 U/mg for SPLA (blue closed circles), and 221 U/mg for SPAH (green triangles). The experiments were performed in triplicates and data are plotted as mean values ± SD

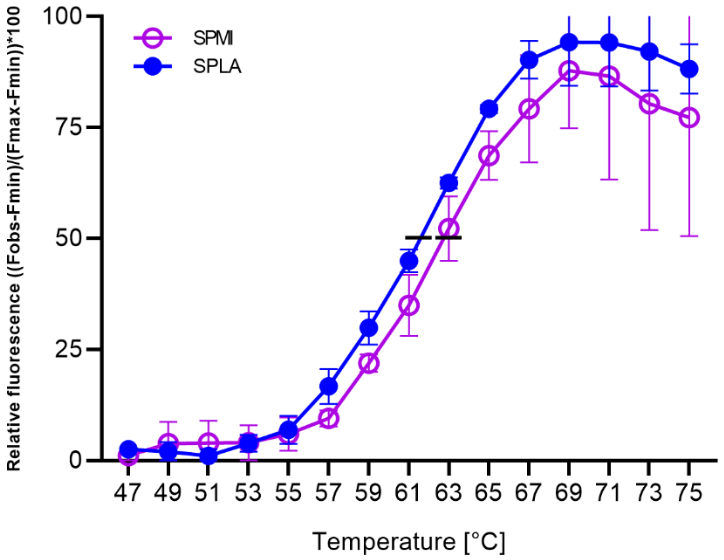


**Fig. S4** Melting curves of purified SPMI and SPLA. The effect of temperature on the stability of the enzyme using SYPRO® Orange as a fluorescence probe, based on the changes in fluorescence emission intensity (Ex/Em = 470/550 nm) (5 x SYPRO® Orange, 10 mM HEPES-NaOH pH 8.0, 3 mM PMSF), is shown as normalized denaturation curves of the thermal shift assay for the proteases SPMI (violet open circles) and SPLA (blue closed circles). The inflection point corresponds to the melting temperature ($T_m$), at which 50 % of the protein is unfolded (-). The experiment was performed in triplicates and data are plotted as mean values ± SD

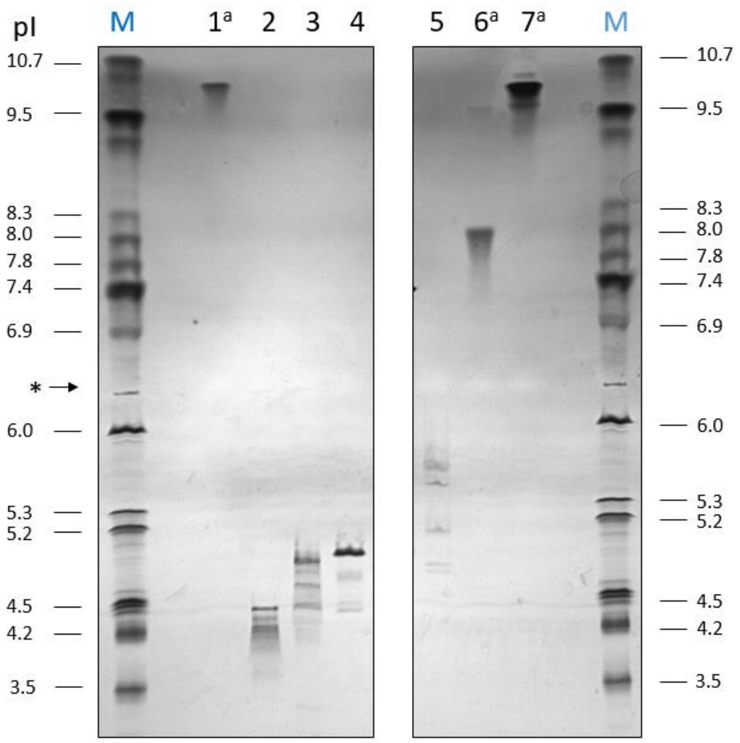


**Fig. S5** Determination of the pI of the purified proteases. Isoelectric focusing was performed with a SERVALYT™ PRECOTES™ wide range pH 3-10 precast gel according to manufacturer recommendations. Lane M, SERVA IEF marker 3-10; lanes 1-7, purified proteases rebuffered in 10 mM HEPES-NaOH pH 7.0. *Sample application point. SPAO (1); SPPM (2); SPAH (3); SPLA (4); SPMI (5); subtilisin Carlsberg (6); Savinase (7). [a] (Falkenberg et al. 2022b)

**Table S1** Oligonucleotides for amplification of the indicated subtilisin genes by PCR using genomic DNA of *P. marinus*, *M. indicus*, *L. alkalitelluris*, and *A. haloalkaliphilus* as template

| Name | Sequence 5’->3’ |
|---|---|
| *aprE_P.marinus_fw* | AGAAGACTTAATGAAAAGATTATTTTTTGCAGTGATG |
| *aprE_P.marinus_rv* | AGAAGACAAGTTAATTCGCTACTTGATAAGTAGTAGT |
| *aprE_M.indicus_fw* | AAAGAAGACGGAATGAAAAAGAAAAAGATTTTCAGTCTGCTTC |
| *aprE_M.indicus_rv* | AAAGAAGACCCGTTATTGAATGGCGGCCTGGAC |
| *aprE_L.alkalitelluris_fw* | AGAAGACTTAATGAAGAAAATGAAATTAGTAAGTAGTATTTTGC |
| *aprE_L.alkalitelluris_rv* | AGAAGACTTGTTACTTTATAGCAGCTTCTACATTAATGAC |
| *aprE_A.haloalkaliphilus_fw* | AGAAGACATAATGAAAAAGCTTCTTATTGTTTTAAGTACC |
| *aprE_A.haloalkaliphilus_rv* | AGAAGACATGTTAGTGAGAGATTGCTTCAAAAGAC |

**Table S2** pI values and number of amino acids in the proteases

| Protease | experimental pI | Number of residues | | | | | AB ratio[a] |
|---|---|---|---|---|---|---|---|
| | | Arg | Asp | Glu | His | Lys | |
| SPPM | 4.3 | 4 | 19 | 8 | 4 | 6 | 1.9 |
| SPMI | 5.5 | 5 | 14 | 6 | 4 | 11 | 1.0 |
| SPLA | 5.0 | 6 | 12 | 9 | 6 | 7 | 1.1 |
| SPAH | 4.9 | 4 | 20 | 9 | 9 | 4 | 1.7 |
| SPAO[b] | 9.8 | 9 | 4 | 4 | 6 | 3 | 0.4 |

[a] The AB ratio [AB = (Glu + Asp)/(Lys + His + Arg)] was calculated as described in (Rhodes et al. 2010).
[b] SPAO from *Alkalihalobacillus okhensis* Kh10-101$^{T}$ (Falkenberg et al. 2022b)
